# Simulation for training in oral cancer biopsy: A surgical model and feedback from GDPs

**DOI:** 10.4317/medoral.17998

**Published:** 2013-02-05

**Authors:** Juan Seoane, Pablo Varela-Centelles, Germán Esparza-Gómez, Rocío Cerero-Lapiedra, Juan M. Seoane-Romero, Pedro Diz

**Affiliations:** 1 Senior Lecturer in Oral Surgery. School of Medicine and Dentistry. University of Santiago de Compostela. Spain; 2 Lecturer. Stomatology Department. School of Medicine and Dentistry. University of Santiago de Compostela. Spain; 3 Senior Lecturer in Oral Medicine. School of Dentistry. Complutense University. Madrid. Spain; 4 PhD student. University of Santiago de Compostela. Spain; 5 Senior Lecturer. School of Medicine and Dentistry. University of Santiago de Compostela. Spain

## Abstract

Objectives: To describe a new bench model for oral precancer/cancer biopsy training and to assess its effectiveness in terms of trainees’ perception. Study design: Cross-sectional, descriptive, performed on 424 general dental practitioners (GDP) who undertook biopsies on a pig tongue. The participants were assessed by direct observation for 2.5 hours using specific check-lists and by means of a self-applied questionnaire. Results: The workshop was perceived as “very interesting” even by those with previous surgical experience (Xi - Xj = 0.07; 95%CI= -0.20-0.09). Most GDPs considered themselves able to undertake oral biopsies on real patients after the workshop. Those who had previously received theoretical continuous education courses on oral biopsy scored higher values within the group (Xi - Xj = 0.20; 95%CI= 0.04-0.37). Conclusions: There is a need for including clinical abilities workshops when instructing on oral biopsy techniques. More studies are needed to validate the procedure and to address cognitive and communication skills.

** Key words:**Models, animal, education, dental, continuing, biopsy, oral cancer, oral surgical procedures.

## Introduction

Oral cancer is a global health problem with increasing incidence and mortality rates (1,2). For most countries, five-year survival rates for tongue, oral cavity and oropharynx cancers are around 50% (1), and this poor prognosis is chiefly related to a late stage of the disease at diagnosis (1,2).

Early diagnosis is critically essential and may have the most impact for improving survival and cure rates (3,4). The standard for detection remains on visual examination and palpation followed by tissue biopsy and histopathological diagnosis (5), being the latter the gold standard for diagnostic procedures and mandatory for every lesion suspicious for malignancy (6), which is paramount for an early detection of oral cancer (7,8).

While some authors advocate for a non-intervention attitude by general dental practitioners (GDPs) when dealing with lesions suspicious for oral cancer or precancer (“no panic, no biopsy, and immediate referral”) (9), others encourage GDPs to biopsy these lesions to assist in the early detection of oral cancer (7) as many studies consider biopsy procedures well within the scope of training and ability for a GDP (6,10). Unfortunately, the number of GDPs who perform oral biopsies, either on a routine or selective bases, is scarce in Europe ( 7% in Turkey (11), 12% in northern Ireland (12), 21% in UK (13), 32% in Spain (14), and 22.7% in Australia (15)), probably due to an undergraduate training mostly focused on theoretical aspects and lack of experience or prac-tical skills in performing biopsy (7,15).

Previous reports have described a wide gap between knowledge on oral cancer diagnosis and professional competence (16) that is reinforced by international data showing most GDPs do not feel competent to undertake oral biopsies (7,14,15) and their self-perceived need for additional training not only on what, where and when to biopsy, but also on when to refer and how to manage the subsequent report (7).

Despite training of skills in simulation laboratories is becoming increasingly common (17) and training of novices in surgical-skills labs leads to improved technical performance in different anatomic sites (18), there are no reports describing a bench model for oral biopsy training, neither the conceptual framework, nor the learning environment and the replication of this surgical situation.

The aim of this study was to describe a new bench model (workshop of abilities) for oral precancer/cancer biopsy training that simulates the surgical environment and to assess its effectiveness in terms of trainees’ perception.

## Material and Methods

A nationwide educational campaign for prevention and early diagnosis of oral cancer was completed by the General Dental Council of Spain (CGOE) in 2010. During this campaign, a cross-sectional pilot study was designed to describe a workshop on clinical abilities for oral precancer/cancer biopsy and to evaluate the trainees’ perception about this bench-model. A total of 424 GDPs volunteered to enter the study and to fill in an anonymous, self-applied, 12-item questionnaire once the workshop was over.

This questionnaire was a modification of a previously used survey instrument (19), which was piloted among a convenient group to ensure practicability. The items were broadly grouped into two sections: profiling questions (demographic and practice), and questions on the trainees’ perceived usefulness of the workshop and on their believed ability to undertake the technique on real patients. The answers had to be graded on a 5-grade Likert-type scale (1 maximum disagreement – 5 maximum agreement).

-Workshop on oral precancer/cancer biopsy clinical abilities

Each participant received a study guide prepared by a panel of experts (“Libro de la biopsia oral”, free access at www.consejodentistas.es). This guide included i) the workshop’s specific objectives, included the Dental Council Referral Scheme (CPG) for lesions suspicious for oral cancer, ii) the Clinical Guide for Early Diagnosis of Oral Cancer, -with indications and contraindications of oral biopsy and information on the technique (theoretical bases of the procedure, methodology and a list of typical errors and complications)-, iii) anatomical details of the animal model to be used in the workshop, iv) a list of the materials required and v) information on the assessment method. This information was also delivered at a seminar at the beginning of the session. In addition, a film demonstrating communication skills by role-playing techniques was projected (available from the CGOE).

The workshop was developed at all 25 dental councils in Spain (including Balearic and Canary Islands), and the participants informed about the conditions of the workshop and safety regulations that were basically identical to those of a real surgical environment. The trainees were divided into pairs and allocated to an adequate scenario within the lab to individually undertake the procedure while the tutors provided intense external feedback to correct potential technique errors in the surgical procedure.

The GDPs were assessed by direct observation during the workshop (2.5 hours) by means of specific checklists that included topics on site selection, amount of tissue removed, specimen handling, use of solutions that stain the surface, time to place the specimen in the fixative provided, specimen identification, and legible and complete paperwork (6). Once the procedure was completed, the trainees were allocated time for autonomous learning.

The study design was approved by the University of Santiago de Compostela Ethics Committee, and the investigation undertaken according to EU ethical protocols.

-Description of the surgical bench model

**Figure 1:**
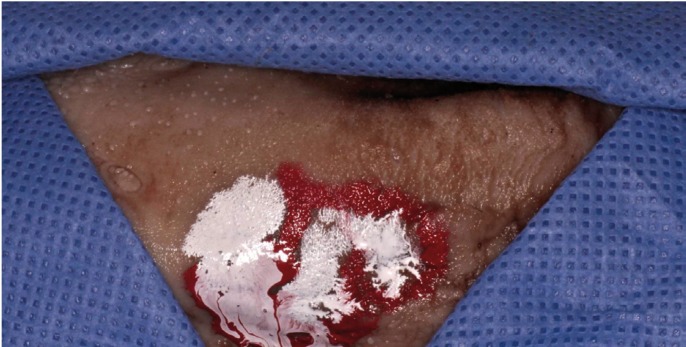
Lesion framed by a fenestrated surgical drape.

The trainees worked in pairs performing the procedures on a pig tongue acting as “operator” or “assistant” consecutively. The 2x3 cm “lesions” were painted on the tongue surface using a number 3 paintbrush (Servian®, Ref: 40 19769 12303 1) and white synthetic enamel paint (Titanlux Esmalte Sintético. Blanco. Cod: 001. Industrias Titan SA. Prat de Llobregat. Spain) to simulate a non-homegeneous lesion on the dorsum-lateral border of the tongue. Occasionally, the “lesion” received some brushstrokes in red colour (Titanlux Esmalte Sintético. Rojo. Cod: 001. Industrias Titan SA. Prat de Llobregat. Spain) to replicate a heterogeneous lesion (Fig. 1). The working area was always framed by a fenestrated surgical drape.

**Figure 2:**
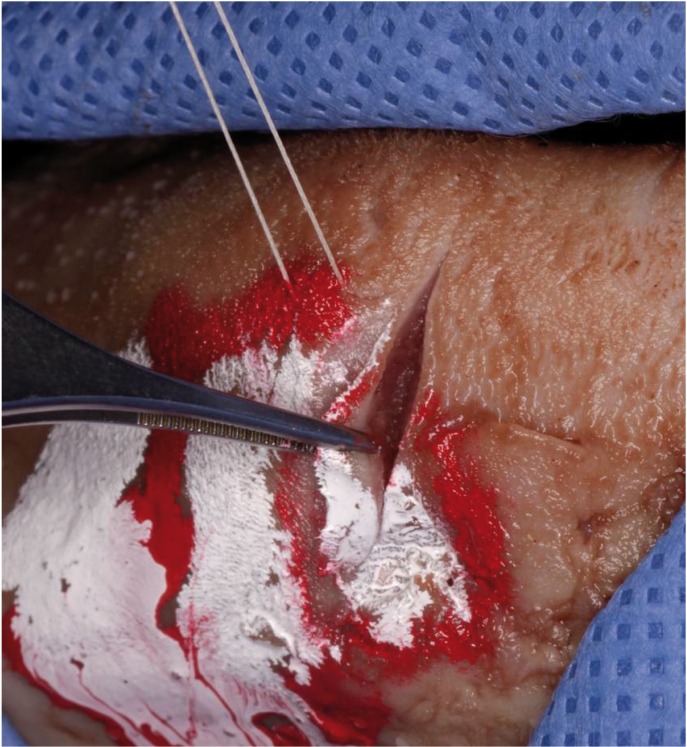
Incisional biopsy on the non-homogeneous lesion.

The participants had to perform an incisional biopsy on the non-homogeneous lesion with the aid of a traction stitch (Fig. 2) choosing the most representative region of the “lesion” (including red, white and healthy areas); the sample obtained should range within 4 to 7 mm long and no deeper than 4 mm. The wound had to be closed with a simple interrupted suture and the sample introduced into a container with an adequate amount of 10% formalin. The trainees had also to write an accompanying report for the Pathology Service including patient data (name, surname, date of birth), medical history (toxic habits, past or present disorders, current medical treatments), information about the lesion (type, number, colour, site, history of the lesion and current symptoms), and type of biopsy performed together with a clinical diagnosis.

-Statistical analysis

Statistical analysis was performed using the SPSS+ 11.0 statistical package (SPSS Inc. Chicago, IL, USA). Data distribution was defined by the mean and the median as central trend statistics, and the standard deviation as spread indicator. The Student’s t test was use for comparing means after assessing their conditions of use and the Pearson’s correlation coefficient to evaluate the association between quantitative variables. The level of significance chosen for all tests was 5%. Confidence intervals around the proportions were calculated using the Epidat 3.1 (Santiago de Compostela, Spain) statistical program.

## Results

-GDPs’ opinions on the oral biopsy workshop

The convenience sample studied included all 424 GDPs from 25 dental councils who attended the onsite course. Their mean age was 37.8±11.0 years, ranging from 23 to 69 (69.1% females; n=293) and had been in practice for an average of 11.8 ±9.0 years (median 10.0 years). Most participants (75.4%) had never attended a continuous education course on this topic (oral biopsy) and 74.7% had never performed a biopsy in a clinical situation.

The workshop was perceived as “very interesting” (mean score 4.5± 0.7; median: 5) even by those GDPs with previous experience on biopsy taking (Xi - Xj = 0.07; 95%CI= -0.20-0.09). The highest agreement rate after the workshop was noted when asked about their ability to perform oral biopsies in a simulated situation (median score: 5) ( Table 1). Most GDPs considered themselves able to undertake oral biopsies on a real patient after completion of the workshop (median score 4.5), although significant differences in terms of self-perceived ability were identified when asked about performing biopsies in different clinical situations (simulation vs real patient) (Xi - Xj = 0.13; 95%CI= 0.02-0.2), as the GDPs felt more capable to perform oral biopsies in a simulated situation than in a real one (p=0.01). It is worth to note that those participants who had received theoretical continuous education courses on oral biopsy before attending this workshop, scored higher values on their perceived ability to undertake biopsies on real patients (Xi - Xj = 0.20; 95%CI= 0.04-0.37). A weak positive association could be established between perceived ability to biopsy oral cancer/precancer lesions and professional experience (r=0.14;p=0.002).

**Table 1:**
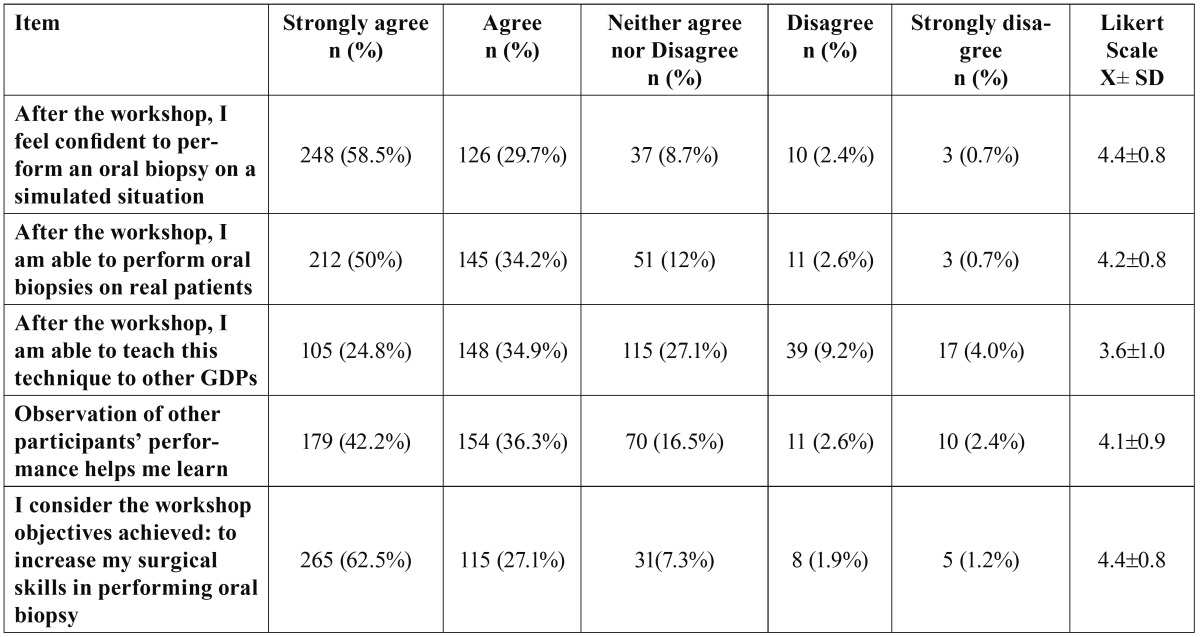
Trainees’ self-assessment of their capacities to undertake an oral biopsy. Survey results.

The trainees also recognized that watching of other colleague’s performance eased learning (X±SD: 4.1±0.9; median: 4) and reported the workshop useful for increasing their practical skills in performing oral biopsies (X±SD: 4.4±0.8; median: 5), particularly for those with previous clinical experience (Xi - Xj = 0.26; 95%CI= 0.11-0.41).

## Discussion

Early cancer lesions may imitate other conditions and often follow an asymptomatic course, which may render them clinically undistinguishable from benign lesions (5,20) and lead to an underestimation of their biological relevance. The implementation of the CPG for referral of lesions suspicious for malignancy and the development of adjunctive aids for visual diagnosis may im-prove the diagnostic sensitivity at primary care level. A low diagnostic specificity would entail oral cancer patients being wrongly referred for a definitive diagnosis, causing an important professional diagnostic delay (21).

Despite being aware of the role of biopsy for an early diagnosis of oral cancer, GDPs still do not feel confident enough to perform biopsies because of different reasons linked to an insufficient knowledge and experience during their undergraduate and postgraduate education (7,15). This finding highlights the need for additional practical experience that fills this educational gap at both undergraduate and postgraduate levels.

Bench models have proved advantageous for surgical training and improving the educational standards (22), particularly when used at early stages of training (23). The classical preceptorship method for teaching manual operative skills has been modified by the recently introduced “clinical abilities laboratories” which offer substantial practical, financial, ethical and theoretical advantages (22,23). Unfortunately, oral biopsy training for oral cancer diagnosis has not been considered under this approach to date.

The bench model proposed in this study is integrated into a simulation that includes cognitive aspects, surgical abilities and communication skills (how to deal with bad news), as simulations in a recreated operation theatre permits teaching and/or assessment of both technical and nontechnical skills (pre-surgical, communication, management of the circumstances…) linked to the par-ticular surgical practice (19,22). This situation may well explain the GDPs’ high interest on the workshop, even those with clinical experience on the topic, who also perceived the workshop as useful for increasing their surgical skills; perhaps because simulation-based surgical training is reported to reduce clinical mistakes and learning curves (22,24).

Previous theoretical knowledge on oral biopsy techniques and the number of years in practice seem to positively influence the trainees’ self-perceived ability to perform oral biopsies on real patients after the completion of the workshop, which agrees with the finding that the number of GDPS who offer biopsy techniques for the diagnosis of oral lesions increases with the number of years of professional experience.

The simulation and the proposed bench model are based on teaching incisional biopsy techniques for early diagnosis of oral cancer, with the exception of obviously malignant lesions that should be urgently referred for specialized care. Although prone to underdiagnosis caused by sampling errors, incisional biopsies may represent a more pragmatic approach for general primary care practitioners (25), as excisional biopsies of malignant lesions performed without oncological criteria may well permit microscopic remnants to stay and destroy the margins of the lesion, making re-excision necessary and, eventually, neck node treatment compulsory.

The participants have considered the workshop helpful for improving their oral biopsy abilities, which supports the dissemination of this educational strategy. Team work and observation of their pair’s performance was reported positive for learning, as has been previously published for similar workshops in diverse surgical specialties, like traumatology, gynaecology and ENT (19,22).

A number of limitations influence our study, namely the difficulty to generalize the results obtained from convenience samples (although convenience samples can provide useful information in pilot studies on a non-previously explored topic), and the participant’s inclination to obtain information on the topic from sources other than the workshop that could not be shared with the rest of the trainees (although more than a half of the participants had never attended a course on oral biopsy). This report also has the limitations inherent to its cross-sectional design, though this kind of studies is valuable for health services policies, to improve clinical practice and to disclose educational gaps.

Within these limitations, our results seem to suggest there is a need for including clinical abilities workshops when instructing on oral biopsy techniques, as it is a supplementary but essential educational resource and supervised clinical practice should always precede autonomous performance on real patients. More studies are needed to validate the procedure and to address cognitive and communication skills, that are clearly essential components of surgical performance.
